# Optimization of production system of shale oil development in Ordos basin, China

**DOI:** 10.1038/s41598-023-33080-8

**Published:** 2023-04-21

**Authors:** Xiaolong Wan, Shuwei Ma, Jianming Fan, Yuanli Zhang, Chao Zhang

**Affiliations:** 1 College of Geosciences, China University of Petroleum (Beijing), Beijing, 102249 China; 2 PetroChina Changqing Oilfield Company, Xi’an, 710018 People’s Republic of China; 3 Research Institute of Exploration and Development, PetroChina Changqing Oilfield Company, Xi’an, 710018 People’s Republic of China; 4 National Engineering Laboratory for Exploration and Development of Low Permeability Oil and Gas Fields, Xi’an, 710018 People’s Republic of China

**Keywords:** Energy science and technology, Engineering

## Abstract

In this paper, production system (PS) of shale oil is optimized according to production data and indoor experiments, including core and fluid tests. Results showed that: ① Pressure drop rate at wellhead is a reasonable reference for the determination of post-fracture shut-in duration (PFSID). When pressure at a wellhead of horizontal well is relatively stable and the pressure drop less than 0.1 MPa per day for three consecutive days, PFSID ends; ② Flowback intensity of fracturing fluid affects the effectiveness of proppant underground, thus flowback intensity can be determined by the critical flow rate and safety factor of each proppant; ③ Flowback intensity should be varied during different development stages, which could be divided into four according to production gas and oil ratio(GOR) of a shale oil horizontal well: low, medium–high, high and high-low production GOR. During the stage of low production GOR, ratio of flow pressure and saturation pressure should be maintained greater than 1.0, and the initial daily liquid productivity for a hundred-meter oil-bearing lateral length in a horizontal well is 2.4 ~ 2.9 m^3^/d; and during the medium–high production GOR, high production GOR and high-low production GOR stages, the responding initial daily liquid productivity should be maintained between 0.8 ~ 1.0 or less than 0.8 respectively.

## Introduction

China's continental shale oil reservoir is greatly different from that of North America in terms of basin size, tectonic environment and sedimentary conditions. Oil-bearing layers in North America are thick and is good in continuity and the shale oil is in the light oil-condensate window, with a high gas-oil ratio and sufficient formation energy. A horizontal well usually achieves a high initial and cumulative production after fracturing and industrialized operation. However, continental shale oil reservoirs in China changes quickly in its planar distribution, and at the same time “sweet” spot selection is difficult. With a low degree of thermal evolution and formation energy, single well production is relatively low. Continental shale strata in China are rich in petroleum resources and were divided into three categories: interlayer, hybrid sediment and shale^1^. Liquid hydrocarbons accumulated or retained in the continental organic-rich shale strata in Ordos basin is typical intro-source unconventional resources, and the reservoirs are interlayer and shale^2–5^. The Ordos basin is located at the junction of the eastern and western tectonic domains of China. It was part of the North China basin during Paleozoic era. The Indosinian movement processed in the Late Triassic caused the Yangtze Plate to squeeze and collide northward with the North China plate, and during the same time the West Qinling orogenic belt uplifted, forming a large asymmetric inland depression lake basin with wide and gentle in northeast while steep and narrow in southwest^6^. Interlayer reservoirs of shale oil are continental clastic sedimentary formation with poor physical properties and complex micro pore structures^7–9^. Porosity of its oil-bearing strata is between 4.0 ~ 12.9%, averaged at 7.4%, and permeability between (0.01 ~ 1.55) × 10^−3^ μm^2^, averaged at 0.1 × 10^−3^ μm^2^^10–12^. After tens of years of shale oil exploration and development practices in Ordos basin, Qingcheng oil field had been built as a demonstration area for a large-scale commercial shale oil development during the year of 2018 ~ 2021, and more than 600 horizontal wells have been in production by the end of 2021, with a well spacing of 300 ~ 450 m and an averaged lateral length of 1650 m. Yearly oil production from interlayer shale oil reservoirs has reached the level of million tons.

Large-scale commercial development of shale oil is aimed to ensure a quick investment return, and the main method is to use ‘quasi-nature’ energy after volume fracturing (VF, a hydraulic fracture with large amount of fluid and proppant to ‘cut’ the reservoir into very small pieces) on a horizontal well. These wells show characteristics of high production rate in its initial recovery stage (the first three months in recovery lifecycle of a horizontal well) and a high decreasing rate. The recovery ratio is relatively low and is able to receive economic benefits under high oil prices while the development can barely sustain under low oil prices. Thus, a reasonable PS aimed to achieve a higher recovery rate is essential for shale oil development under low oil prices, other than “sweet spot” selection, optimization of well layout and engineering technologies, and reduction in investment. A reasonable PS of a horizontal well should achieve a smooth transition of different driven energies while reduce formation damage applied by fracturing liquid^13^. An overall shale oil development process can be divided into three stages: post-fracture shut-in stage, drainage stage and recovery stage. Thus a reasonable PS should be suitable to different stages: to optimize the PFSID to maximize energy liberation imposed by hydraulic fracturing while reduce formation damage; to certify a reasonable flowback intensity of fracturing liquid, since an unsuitable flowback intensity may cause sand spit from a fractured reservoir, while a suitable intensity helps the closure of fractured cracks and is of important to energy maintenance during later oil recovery stages and to reduce decreasing rate of a horizontal well; to optimize fluid recovery intensity during recovery stages, during which an effective usage of formation energy reduces the decreasing rate during initial stage. Elastic energy imposed by fracturing liquid is releasing during production stage, and a high liquid productivity in this stage leads to the release of dissolved gas and dissolved gas drive appears during this stage, resulting in a two-phase flow of oil and gas and a high decreasing rate. Thus, a reasonable productivity of a horizontal well ensures an orderly release of three kind of elastic energies underground: energy imposed by fracturing liquids pumped, by deformation of formation rocks and liquids, and by dissolved gas. For continental shale oil development in China, in-situ practice is ahead of theory research, and this paper aims to find solutions to the problems encountered during shale oil development, which are mentioned above. And the following three sections are the solutions to each of the problems above. In this paper, a series of reasonable PS for horizontal wells were determined based on theoretical analysis and in-situ practices, aiming to improve individual well productivity during initial stage and estimated ultimate recovery (EUR).

## Optimization of post-fracture shut-in duration

The overall wettability of shale oil reservoir in Ordos basin is neutral or weak hydrophilic. Bottom hole pressure (BHP) of a horizontal well is much higher than initial formation pressure (IFP) after a large-scale VF, and it accelerates liquid exchange between well bore and reservoir, where oil underground is displaced by fracture liquid ‘imbibed’ into the reservoir matrix. Post-fracture shut-in ends when BHP and IFP reaches a balance.

### Wettability of Chang 7 shale oil reservoir

Reservoir wettability is a key factor affecting water imbibition efficiency underground, and a hydrophilic reservoir shows a higher oil displacement efficiency^5^. Eighteen core samples were collected to test reservoir wettability by self-absorption method. The overall wettability is neutral to weak hydrophilic (Table 1).

**Table 1:** Results of wettability experiment from 18 core samples.

Wettability index			Wettability
Oil wettability index	Water wettability index	Relative wettability index	
0.57	0.83	0.26	Neutral–weak hydrophilic

### Mechanism of oil imbibition displacement

In porous media, fracture liquid is imbibed into hydrophilic oil-bearing reservoir displacing oil from matrix, and fracture liquid movement is affected by capillary pressure and gravity^10^. During this process, oil flows from reservoir matrix to the fractured cracks and fracturing liquid into small pores in reservoir. Previous research from reverse imbibition and water huff and puff experiments showed that for a core sample with 0.2 × 10^−3^ μm^2^ permeability, inflection point of its imbibition distance appears as 7.6 cm, and this is a very short imbibition distance^14–16^. During in-situ practices of post-fracture shut-in process of shale oil development, imbibition only takes place where the fracturing liquid reach, and the sweep volume is then a key factor affecting shale oil recovery.

**Figure 1:**
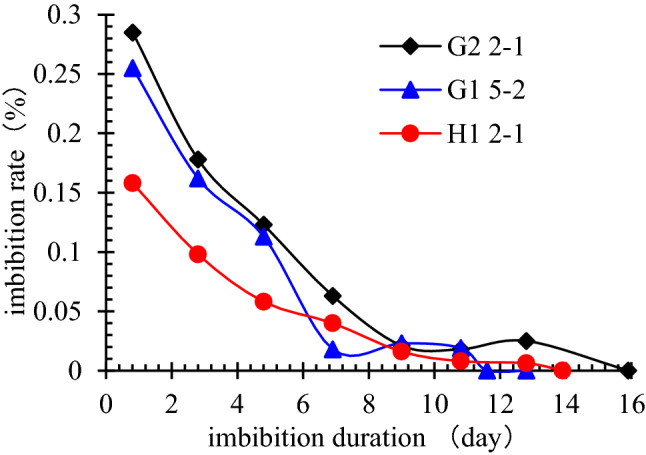
Imbibition velocity of different cores.

**Figure 2:**
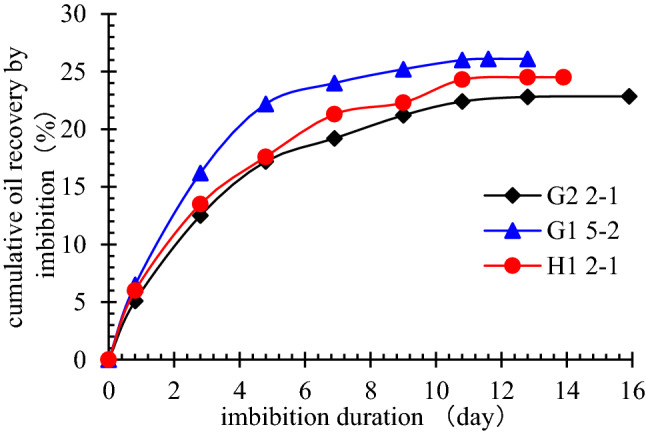
Cumulative imbibition recovery of different cores.

Relative recovery for core samples with different physical properties showed that imbibition mainly happens in medium and small pores (Table 2), and curve of the process shows a “two-step” shape: the first step is called “fast-velocity” period, the imbibition velocity shows a fast decrease rate during initial period, and the cumulative oil recovery by imbibition increases sharply; the second step is “steady” period, inflection point appears at 7 days when change of imbibition velocity and cumulative recovery curves becomes gentle (Figs. 1 and 2).

**Table 2:** Relative recovery of different pores of Chang 7 formation.

Core number	Porosity/%	Permeability/ × 10^−3^ μm^2^	Oil recovered from pores with different radius/%		
			Less than 10 μm	10 ~ 20 μm	Higher than 20 μm
H1 2–1	8.75	0.049	64.69	33.03	2.25
G1 5–2	9.94	0.084	39.89	48.23	11.88
G2 2–1	10.58	0.097	39.20	47.99	12.81

### Impact of PFSID on flowability of crude oil

**Figure 3:**
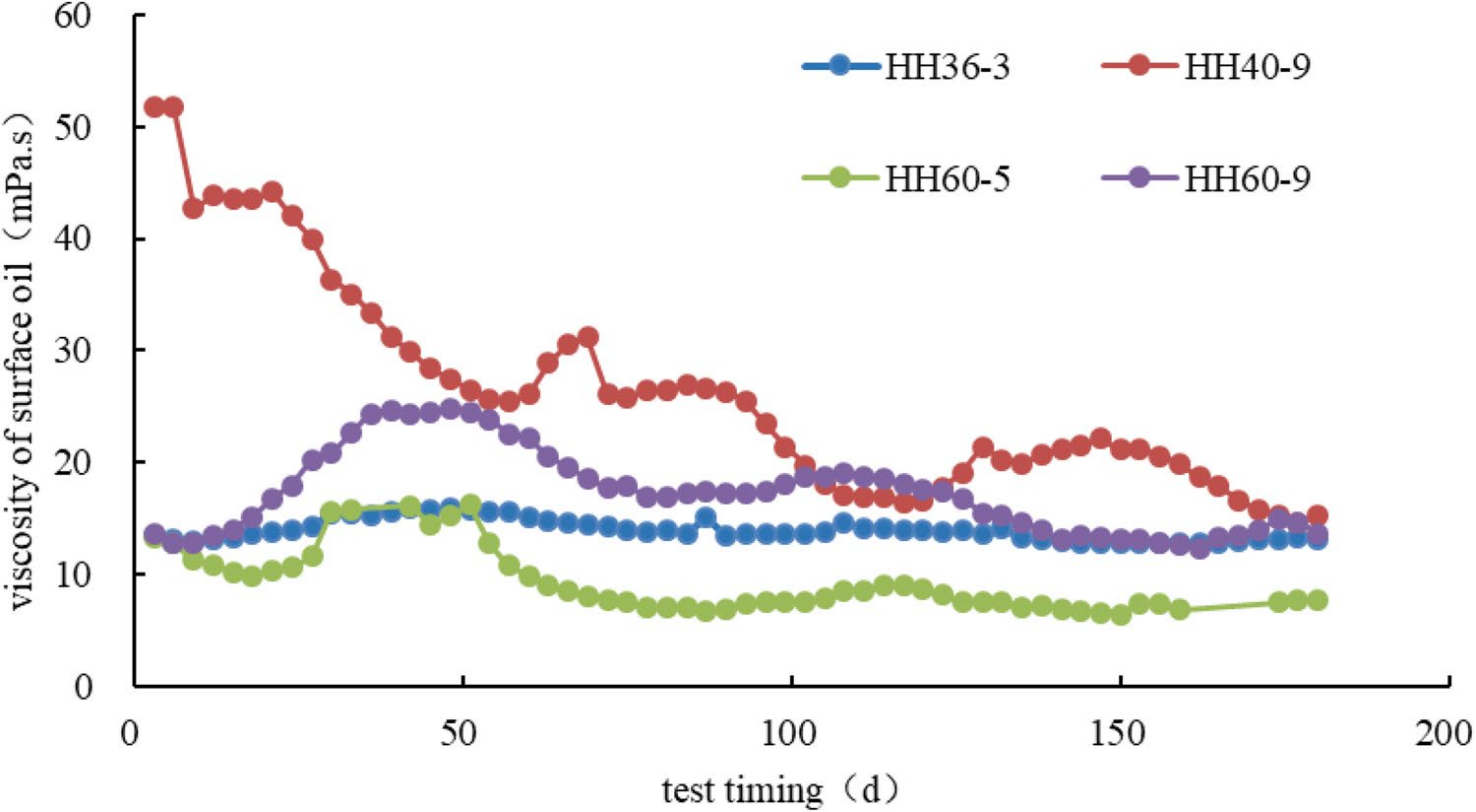
Viscosity of emulsified crude oil from 4 horizontal wells under formation temperature (60 °C) and atmospheric pressure.

To exam the impact of fracturing liquid on flowability of crude oil, we analyzed oil viscosity at surface collected from 4 horizontal wells in Qingcheng oil field with 135 days of averaged PFSID. The experiment was conducted under formation temperature (60 °C) and atmospheric pressure. Results showed that the average viscosity of oil samples were 12.5 mPa.s, three time higher than viscosity of normal crude oil on the surface (4.0 mPa·s) (Fig. 3), meaning that during post-fracture shut-in period, oil shows emulsification leading to a higher viscosity and lower flowability of crude oil. The PFSID should be maintained in a reasonable window.

### Optimization of PFSID

**Figure 4:**
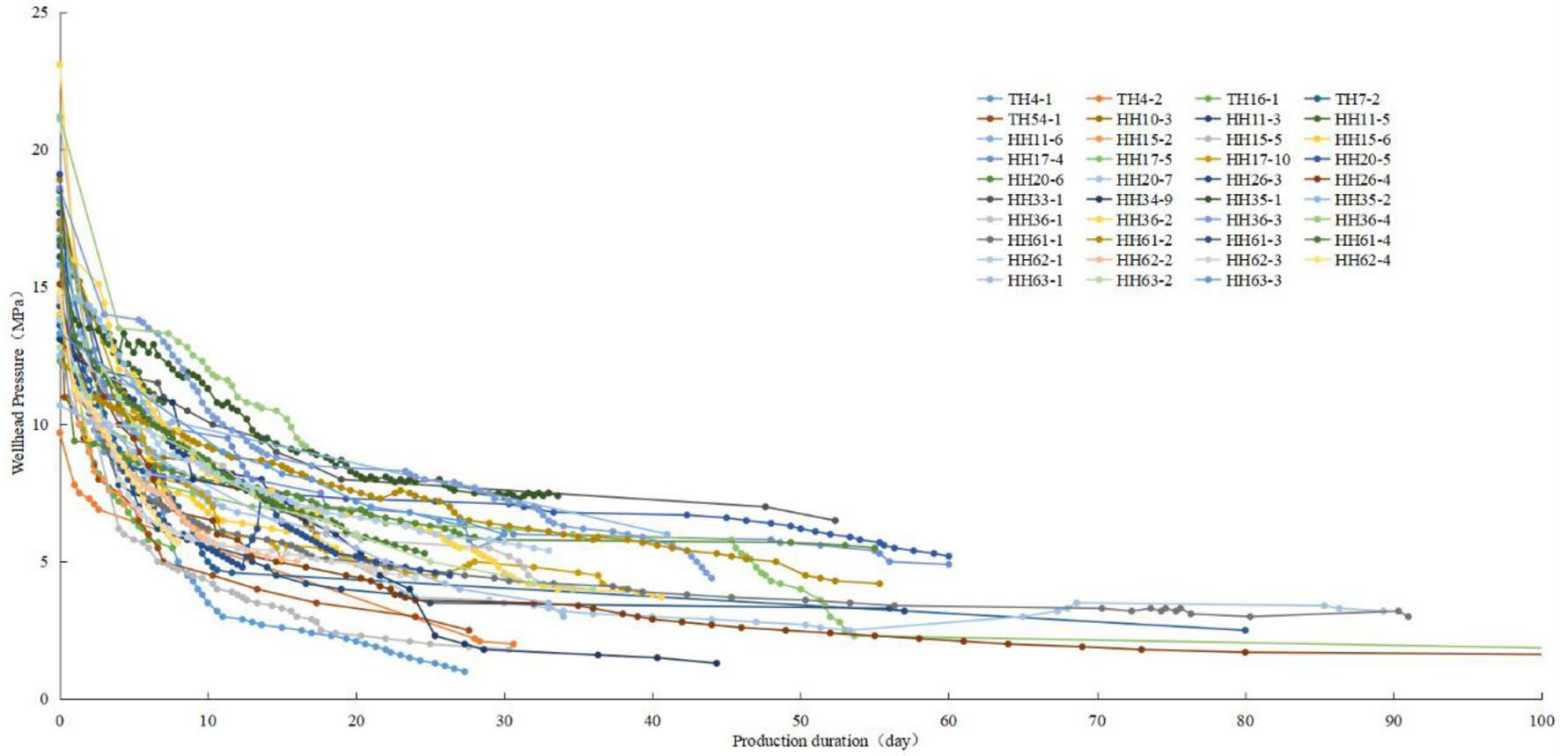
Wellhead pressure drop curve with time of horizontal well in interlayer shale oil reservoir.

**Figure 5:**
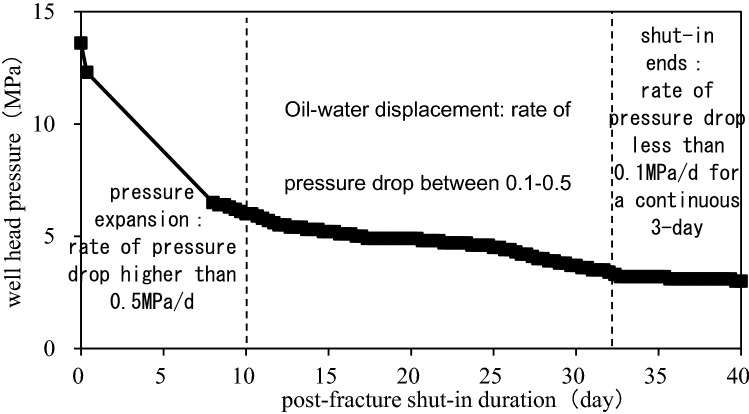
Division of well shut-in stages using wellhead pressure drop.

Except for the experiments conducted above, we also found that PFSID could be determined by BHP. BHP is usually calculated by wellhead pressure which is measured by piezometer installed at wellhead after hydraulic fracture. Shape of pressure drop curve shows two stages (Fig. 4): fast drop stage (pressure drop rate higher than 0.5 MPa/d) and slow drop stage (pressure drop rate between 0.1 and 0.5 MPa/d), and they correspond to “fast-velocity” period and “steady” period of the imbibition process mentioned in 2.2. Thus, considering imbibition distance, imbibition stage and the flowability of the fluid, we use pressure drop rate at wellhead to divide different PFSI stages and to determine when PFSID ends. When the pressure drop rate is higher than 0.5 MPa/d, BHP increased by fracturing fluid is expanding from wellbore to reservoir matrix; when the pressure drop rate is between 0.1 ~ 0.5 MPa/d, oil–water displacement is considered to be taken place; and the well shut-in ends when the pressure drop rate lower than 0.1 MPa/d for a consecutive three days (Fig. 5). The whole process usually maintains less than 30 days.

## Optimization of flowback intensity during drainage stage

Horizontal well is in its drainage stage after PFSI and when salinity of produced water equals to that of initial formation water, the stage ends. Both the salinity of produced water and formation water are able to be obtained from indoor experiment, and salinity of formation water can also be obtained from exploration wells, appraisal wells and structure wells that have been producing for more than two years. Production data from Qingcheng oil field shows that the average drainage rate of horizontal wells (ratio of water produced both during oil testing and development stage and total fracturing liquid) is 13.5%.

### The end of drainage stage

**Figure 6:**
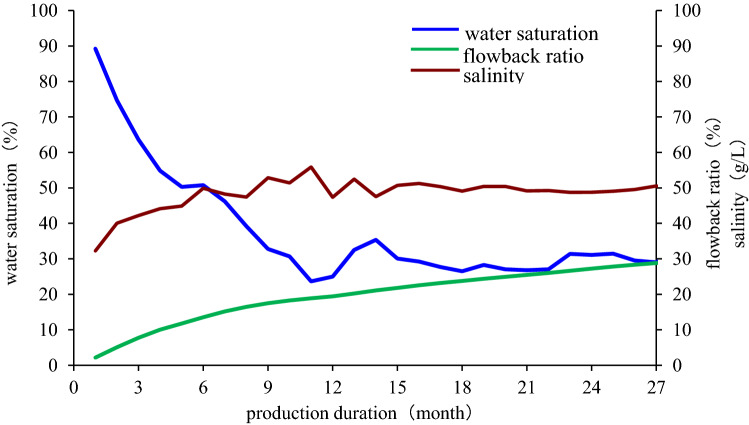
Curve of water saturation, salinity and flowback rate in Qingcheng area.

Salinity of formation water in Qingcheng area is 53.9 g/L. Salinity of produced liquid increases with it flowing from underground and will equal to that of formation water eventually. Increase of salinity slows when water saturation of the produced liquid drop to 60% and this is the time when salinity of produced liquid equals to that of formation water. The curve of salinity becomes steady when water saturation is between 75 and 50%, and maintains at around 50 g/L after a little fluctuation. From our in-situ practices and for our convenience of evaluating all the horizontal wells, we usually use water saturation of 60% as the point where the drainage stage ends. Thus, drainage stage ends when water saturation drops to 60% (Fig. 6).

### Determination of flowback intensity

An aggressive flowback strategy is able to speed up the productivity in the early lifecycle of a well and have minimal impact on the long-term performance of a well with modern completion when not considering cracks and formation damage^17^. A reasonable flowback strategy is determined by critical flow rate of proppant and is able to be calculated by critical flow formula.

The critical flow rate is the moment when proppant sand starts to move underground:

1 $$ V_{c} = \frac{1}{3}\sqrt {\frac{{2\sqrt 3 {\text{g}}d_{s} \left( {\rho_{s} - \rho } \right)}}{\rho }} $$

where: ρ_s_—proppant density, kg/m^3^;ρ—Fracturing fluid density, kg/m^3^;d_s_—diameter of proppant sand, mm;g—gravitational acceleration, m/s^2^;V_c_—critical flow rate, m/s.

We assumed that the shape of fractured cracks is rectangle and the critical flow volume is calculated by the following:

2 $$ Q_{C} = 1.5 \times {\text{N}} \times 10^{2} h_{f} w_{f} V_{c} \phi_{{\text{p}}} /B_{o} $$

where: Q_c_—critical flow volume, m^3^/h;N—number of effective clusters;h_f_—reservoir thickness, m;w_f_—width of the fractured cracks, m;Φ_p_—plane porosity of the propped fracture, %;B_o_—volume factor.

**Figure 7:**
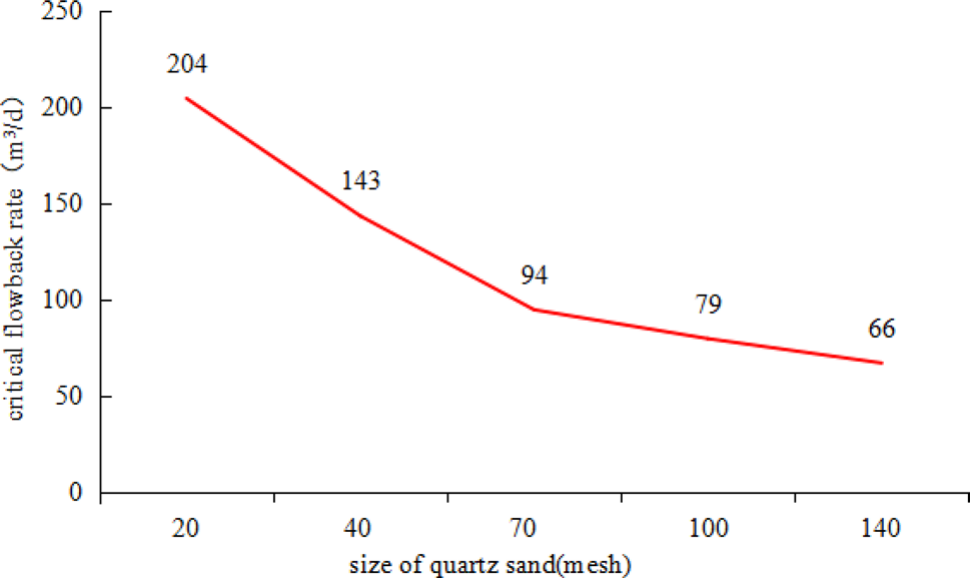
Relationship between critical flowback rate and size of quartz sand.

The average thickness of shale oil reservoir in Qingcheng area of Ordos basin is 15 m, and size of the quartz sand used as proppant during fracturing is between 40  and 70 mesh. Porosity and width of the propped fracture is 20% and 0.01 m. Volume factor of water-base fracturing fluid is 1.1. We drew a chart about the proppant sizes and critical flowback rate (Fig. 7). With the size of quartz sand less than 70 mesh, the critical flowback rate is less than 95 m^3^/d. We add a safety factor of 0.85 ~ 0.90 when use this chart to determine critical flowback rate under in-situ practices—flowback intensity of a horizontal is the critical flowback rate times safety factor.

### Applications

Porosity of Chang 7 shale oil reservoir of Qingcheng area is 8% with oil saturation of 71% and reservoir thickness of 16 m. Ten horizontal wells (HP1 ~ 10) were deployed in this area with a lateral length of 1500 m and well space of 1000 m. The calculated flowback intensity of well HP1 ~ 5 is 500 ~ 1000 m^3^/d, and these wells showed very sever sand splitting in wellbore during flowback, while flowback intensity of 80 ~ 100 m^3^/d was applied for well HP6 ~ 9 and showed no sand splitting. Here, well HP7 is taken as an example, on which segmented multi-cluster fracturing with open hole packer was applied. The well was fractured for 12 stages with 7352.2 m^3^ of fracturing liquid. Proppant size is 70 mesh and with a density of 1410 kg/m^3^. Density of fracturing liquid is 1000 kg/m^3^ and diameter of proppant sand is 0.000212 m. Plane porosity of the propped fracture is 20% and volume factor 1.1. The calculated critical flowback volume is 95 m^3^/d and flowback intensity 80 ~ 86 m^3^/d. The in-situ flowback intensity is 85 ~ 92 m^3^/d, and produced 1227.2 m^3^ of fluid in 14 days. The well was put into production in Nov. 2013 and 28 thousand tons of oil has been recovered by Dec. 2021, the EUR is 9.4% and the final cumulative oil is estimated 51.7 thousand tons.

## Optimization of development system in different recovery stages

**Figure 8:**
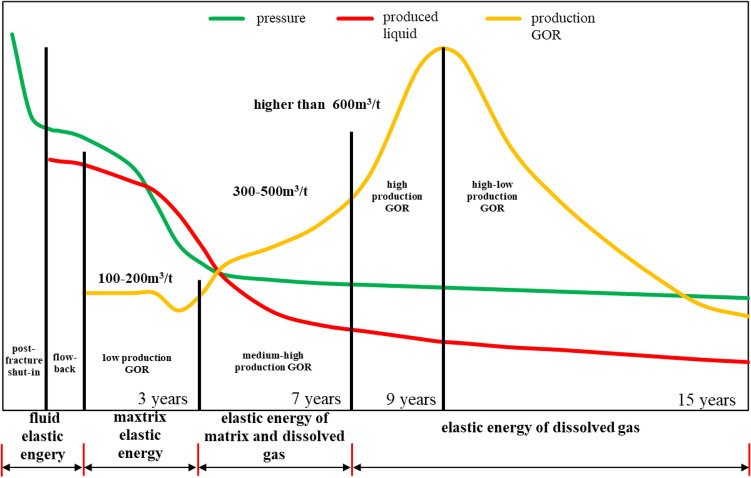
Division of quasi-natural energy development stages of shale oil horizontal wells with large-scale volume fracturing.

To fully use the energy of dissolved gas drive, a full-life cycle of shale oil recovery can be divided into four stages according to production GOR, referring to waterflooding reservoir: low, medium–high, high and high-low production GOR (Fig. 8). The primary consideration during shale oil development is how to fully use formation energy underground, thus relationship between GOR, flowing pressure and oil productivity is analyzed to optimize liquid intensity during different recovery stages.

### Relationship between GOR, ratio of flowing pressure and saturation pressure (RFS) and productivity

One hundred and nineteen wells in Qingcheng area that have been producing continuously for more than one and a half years were selected to study production performance of shale oil horizontal wells. The average lateral length of these wells is 1695 m with an 73.2% of oil-bearing lateral length and 11.0% of tested whole hydrocarbon. They were fractured for 22 stages and 107 clusters, and an average of 28,808 m^3^ liquid and 3219 m^3^ sand were pumped underground each well. The average initial daily oil productivity is 15.9 t/d and current daily productivity is 9.7 t/d.

**Figure 9:**
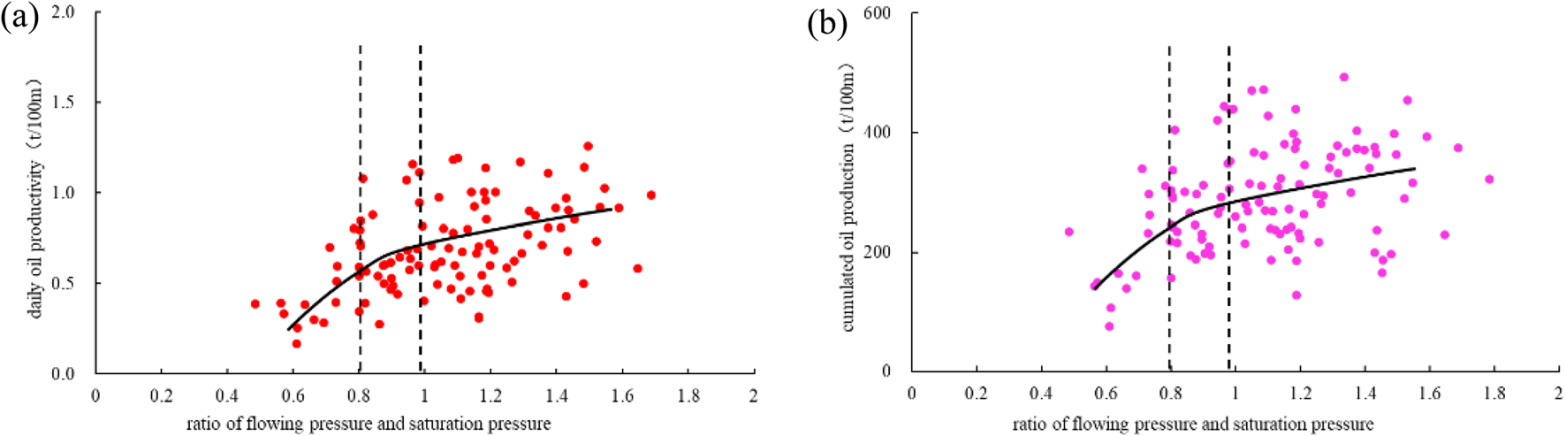
(**a**) relationship between RFS and daily oil production per hundred-meter-meter of oil-bearing formation (**b**) relationship between flow saturation ratio and cumulative oil production for a hundred- meter oil-bearing lateral length.

We found that drop of GOR starts to accelerate when flowing pressure is lower than 80% of saturation pressure (Fig. 9). RFS is positively correlated to both well productivity per hundred-meter oil-bearing lateral length and cumulative oil production. The curve of the correlation shows a “three-stage” characteristics: RFS shows no obvious correlation with well productivity when it is higher than 1; well productivity starts to decrease with the drop of RFS and the drop trend accelerates when RFS is lower than 0.8. For wells with low RFS, very serious degassing appears and the low usage of formation energy results in a poor development of shale oil (Fig. 9a,b).

### Optimization of liquid productivity during different recovery stages

**Figure 10:**
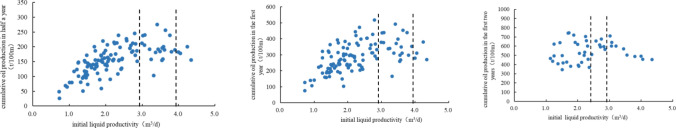
Relationship between initial liquid productivity and cumulative oil production per hundred-meter oil-bearing lateral length in different periods.

Figure 10 shows a relationship between initial liquid productivity and cumulative oil production from per hundred-meter oil-bearing lateral length from horizontal well in different periods. Liquid productivity during low GOR stage is able to be optimized by analyzing initial liquid productivity and cumulative oil production per hundred-meter oil-bearing lateral length. We found that during low GOR stage liquid productivity should be maintained between 3.0 ~ 4.0 m^3^/d for a hundred-meter oil-bearing lateral length to receive a higher productivity during initial stage, and that between 2.4 m^3^/d and 2.9 m^3^/d to have a higher cumulative oil production in later stages (Fig. 10).

**Figure 11:**
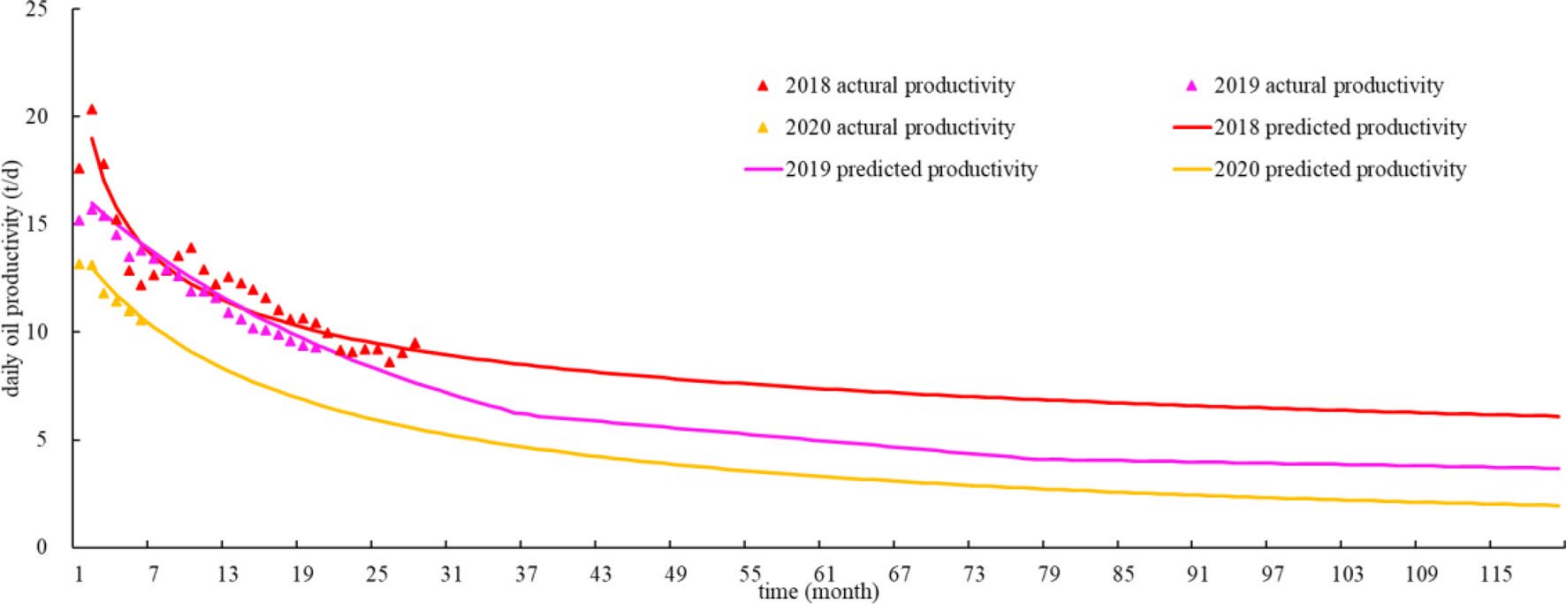
Fitting curve of yearly production drop of horizontal wells in Qingcheng area.

**Figure 12:**
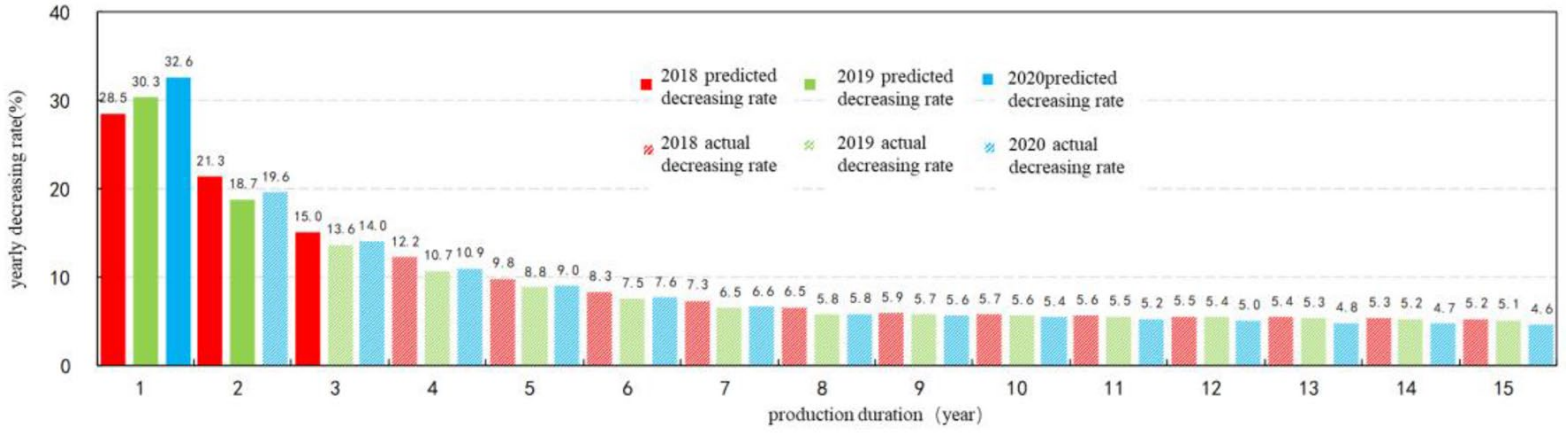
Yearly production decrease shale oil horizontal wells in Ordos Basin.

Liquid productivity in medium–high, high and high-low production GOR stages could be optimized by the predication of oil production. Decreasing characteristics of the oil production in Qingcheng area is similar to Alps hyperbolic decline (Fig. 11)^18^. Data fitting of production trend showed that production drop rate varies for wells that put into production in years of 2018, 2019 and 2020. For wells of 2018, the drop rates of the first three years are 28.5%, 21.3% and 15.0%; and that of 30.3%, 18.7%, 13.6% for wells of 2019 and 32.6%, 19.6%, 14.0% for wells of 2020. Differences in these drop rates is less than 1% in their first five production years (Fig. 12). Thus, for wells in their medium–high, high and high-low production GOR stages, the reasonable liquid productivity can be calculated by predicted drop rate.

### Understanding of development characteristics during different stages

Low production GOR stage: GOR is between 100 m^3^/t and 200 m^3^/t. During this stage, energy increased by pumped liquid, from reservoir deformation and from underground fluid, as well as expansion energy of dissolved gas should be fully used. Expansion energy of dissolved gas comes from gas expanding in the oil and yet is not starting to flow. This energy is able to drive oil to the bottom of a well. During this stage, FSR is higher than 1.0. Initial liquid productivity is between 2.0 m^3^/d and 2.5 m^3^/d for a hundred-meter oil-bearing lateral length. And this stage is predicted to be maintained for 3 years and methods should be applied to prolong this stage to increase ultimate oil recovery.

Medium–high production GOR: GOR is between 200 m^3^/t and 600 m^3^/t. This is the early stage of dissolved gas drive. Gas compressibility factor is much higher than comprehensive compressibility factor, then elastic expansion energy of dissolved gas is the main drive force during this stage. Flowing pressure decreases with the drop of formation pressure. In this stage, RFS is kept between 0.8 and 1.0 to maintain a certain liquid intensity.

High production GOR: GOR is higher than 600 m^3^/t and the well is in its medium-later stage of dissolved gas drive with RFS lower than 0.8. In this stage, the fluid underground is highly degassed and oil viscosity increases. Flowability decreases and the production GOR is 6 times higher than initial GOR.

High-low production GOR. This is the late stage of dissolved gas drive. During this stage, formation energy has been highly consumed and both liquid productivity and production GOR is continuously decreasing, and the well will end up in producing neither gas nor liquid.

### Applications

**Figure 13:**
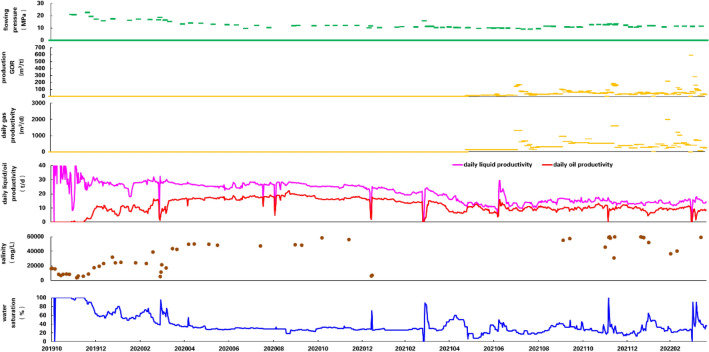
Production curve of well HH11-1.

Shale oil development with horizontal wells in Qingcheng has started from 2018 and gained much experiences since then. We selected HH11-1 as the typical well because its geological conditions and fracturing scale is close to the average level of the area, and it has been producing for a relatively long period. Its lateral length is 1719 m with 972 m of oil-bearing lateral length and 195 m of inferior oil-bearing lateral length. The well is fractured for 22 stages and 98 clusters with 4285 m^3^ sand and 30,377 m^3^ liquid pumped underground. Fracturing sand ratio is 18.5% and the treatment volume is 11.2 m^3^/min. Daily liquid and oil productivity during initial stage are 29.1 m^3^ and 20.0 t respectively, and liquid flowback intensity is 2.5 m^3^/d for per hundred-meter lateral length. The FSR is maintained higher than 1.0 (BHP is 10 MPa). This well has been producing for more than three years and the current daily liquid and oil productivity are 17.2 m^3^/d and 11.6 m^3^/d. The cumulative oil production is 18,933 t and the well shows a good development result (Fig. 13).

## Conclusions

1. Shale oil reservoir of Ordos basin is neutral or weak hydrophilic, which favors to oil imbibition displacement; this process mainly takes place in medium-small pores in matrix and the curve shows a “two-step” shape: fast-velocity and slow-velocity period; the longer the post-fracture shut-in duration, the more possible oil gets emulsified and flowability worsened. PFSID is determined by pressure change at wellhead. Change of pressure can be divided into 3 stages and its decreasing rate slows down after 30 days of PFSID. By analysis we mentioned in this paper, we proposed that a reasonable shut-in duration should be divided by the pressure drop rate, and the duration ends in 30 days.
2. Sixty percent of water saturation marks the end of flowback process, and that is the moment when salinity of flowback liquid is equal to that of formation water. Flowback intensity can be calculated by the critical flowback rate of the proppant times safety factor. For physical characteristics of shale oil reservoir in Ordos basin, we have formed a chart showing relationship between critical flow rate and proppant sizes, and it helps to determine the flowback intensity of a horizontal well.
3. The whole development life-cycle of a horizontal well can be divided into four stages according the change of production GOR: low, medium–high, high and high-low production GOR;
4. Flowback intensity during different development stages can be optimized according to relationships between production GOR, flowing pressure and productivity. Flowback intensity during low production GOR stage can be optimized by liquid productivity and cumulative production per hundred-meter oil-bearing lateral length, and that during medium–high, high and high-low production GOR can be optimized by predicted drop rate. For horizontal wells in Qingcheng oilfield, flowback intensity should be maintained between 3.0 ~ 4.0 m^3^/d for a hundred oil-bearing lateral length during initial stage and 2.4 ~ 2.9 m^3^/d during later production stage.

## Supplementary Information

Supplementary Information.

## Data Availability

All data generated or analysed during this study are included in this published article [and its supplementary information files].
